# The intake of ultra-processed foods and homocysteine levels in women with(out) overweight and obesity: The Rotterdam Periconceptional Cohort

**DOI:** 10.1007/s00394-024-03334-w

**Published:** 2024-02-21

**Authors:** Nicole Schenkelaars, Lenie van Rossem, Sten P. Willemsen, Marijke M. Faas, Sam Schoenmakers, Régine P. M. Steegers-Theunissen

**Affiliations:** 1 Department of Obstetrics and Gynecology, Erasmus MC, University Medical Center, Dr. Molewaterplein 40, 3015 GD Rotterdam, The Netherlands; 2 Department of Biostatistics, Erasmus MC, University Medical Center, Dr. Molewaterplein 40, 3000 CA Rotterdam, The Netherlands; 3 Department of Pathology and Medical Biology, University of Groningen and University Medical Center Groningen, Groningen, The Netherlands

**Keywords:** Pregnancy, Ultra-processed foods, Homocysteine, Obesity, 1-CM metabolism

## Abstract

**Purpose:**

Today’s diet consists of a substantial proportion of ultra-processed foods (UPF), especially in women with overweight and obesity in the reproductive period. High UPF intake results in an inadequate and unbalanced diet leading to derangements of several metabolic pathways detrimental to pregnancy and birth outcomes. Therefore, we aim to investigate whether UPF intake in the periconceptional period affects total homocysteine plasma levels (tHcy).

**Methods:**

1532 participants were included from the prospective Rotterdam Periconceptional Cohort. UPF intake was calculated using Food Frequency Questionnaires including items classified as 4 in the Nova classification, and tHcy was measured by using liquid chromatography-tandem mass spectrometry system, with an interassay coefficient of variation of < 5.5%. Multivariable linear regression modeling was used and adjusted for covariates and significant interaction terms.

**Results:**

Women with overweight or obesity showed significantly higher percentage of UPF intake (respectively, 50.3 and 51.3%) and higher tHcy (respectively, 6.6 and 6.3 µmol/L, Kruskal–Wallis test; respectively, *p* < 0.001 and *p* = 0.04) compared to women with normal BMI (UPF intake: 46.8%, tHcy: 6.1 µmol/L). A 10% higher intake of UPF was associated with an increase in tHcy (adjusted: *β* = 1.31, 95% CI = 0.38–2.23). Analysis stratified for BMI classification showed comparable associations in normal weight participants (adjusted: *β* = 1.07, 95% CI = 0.06–2.07); however, no significant association in participants with overweight (adjusted: *β* = 0.06, 95% CI = − 0.95–1.07) and obesity (adjusted: *β* = 1.70, 95% CI = − 0.52–3.92) was shown.

**Conclusion:**

This study showed that a higher intake of UPF is associated with increased tHcy. Better knowledge and awareness of the nutritional quality of the diet in the periconceptional period may contribute to 1-CM and subsequently improve pregnancy course and outcome.

**Trial registration number and date:**

NTR4356, November 2010.

**Supplementary Information:**

The online version contains supplementary material available at 10.1007/s00394-024-03334-w.

## Introduction

During the periconception period, the mother’s nutrition status is of great importance, because it is a major determinant of fertility, embryonic, and fetal development [1–3]. However, following the Developmental Origins of Health and Disease hypothesis, the maternal nutrition status is even more crucial before conception, as it affects genomic imprinting and subsequently programs the child’s long-term health [4]. A suggested underlying mechanism of this theory includes epigenetic modifications, which can alter gene expression without the mediation of changes in DNA sequence, such as DNA methylation. One-carbon metabolism (1-CM) is crucial in this mechanism by providing the methyl groups (1-CM units) required for DNA and RNA synthesis and methylation [5, 6].

**Fig. 1:**
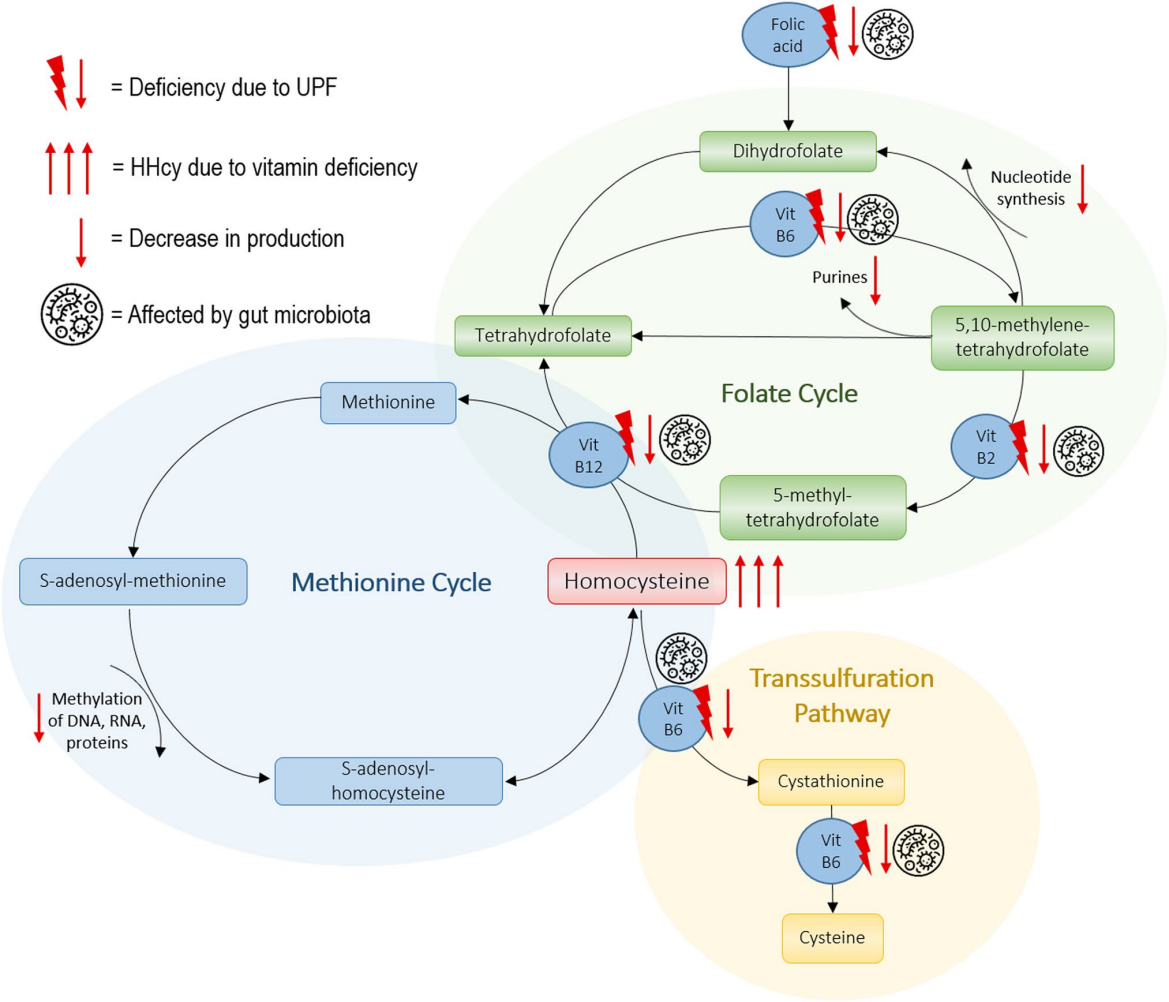
Hypothetical pathway of disturbances in 1-Carbon Metabolism and gut microbiota due to excessive ultra-processed food intake (2, 12–14)

Homocysteine is derived as an intermediate product in the biosynthesis of cysteine (Cys) and methionine (Met) as part of 1-CM (Fig. 1). Low levels of B-vitamins, including folate, derange 1-CM, resulting in increased homocysteine levels or hyperhomocysteinemia (HHcy; total homocysteine plasma level (tHcy) > 15umol/L). HHcy is involved in the causative pathway of pregnancy complications developing in the periconceptional period, including neural tube defects, recurrent miscarriages, and adverse late pregnancy outcomes such as pregnancy-induced hypertension and pre-eclampsia [7–9]. Furthermore, studies are showing that HHcy is associated with lifestyle-related risk factors including smoking, alcohol, fruit and vegetable intake, and having overweight or obesity [10, 11].

With approximately 40% of all adults worldwide having overweight or obesity, the aforementioned pregnancy complications, cause a serious risk for these women, and therefore, prevention is of utmost importance [12]. Contributing to the increasing prevalence of obesity is the wide availability of cheap unhealthy foods, such as ultra-processed foods (UPF) [13, 14]. Compared to less processed foods, UPFs are different in composition, as they typically have more calories and greater quantities of sugar, salt, and saturated fat. Foods high in saturated fats and simple carbohydrates are generally linked with worse general health effects, and micronutrient deficiencies [15].

Additionally, increased consumption of simple sugars and fats is directly linked to increased levels of intracellular prooxidants and alterations in the gut microbiota composition and their functions, leading to an imbalanced composition of gut bacteria, which is also observed in people with obesity [16, 17]. The gut microbiota and the 1-CM are strongly interconnected, because the dysbiotic state of gut microbiota impairs the ability of nutrient absorption from the gut and the synthesis of 1-CM moieties [18]. This indirectly contributes to the impaired 1-CM, with consequently elevated tHcy.

In the present study, we aim to investigate the hypothesis that a diet high in UPF will directly lead to a shortage of micronutrients, substrates, and co-factors of the 1-CM. Moreover, indirectly it leads to gut dysbiosis, resulting in an impaired absorption and synthesis of 1-CM moieties and subsequently increased tHcy (Fig. 1). Moreover, we investigate the subpopulation having an increased BMI from the assumption that a chronic inflammatory body state may affect our hypothesis. This will address the knowledge gaps to what extent UPF intake has a direct effect on 1-CM, and the relevance of tHcy as a (clinical) measurable end-product and biomarker of a poor nutrient diet or unhealthy lifestyle. Confirming our hypothesis would allow for monitoring of periconceptional women to prevent HHcy and improve pregnancy and birth outcomes by informing these women to refrain from high intake of UPFs.

## Methods

### Study population and design

**Fig. 2:**
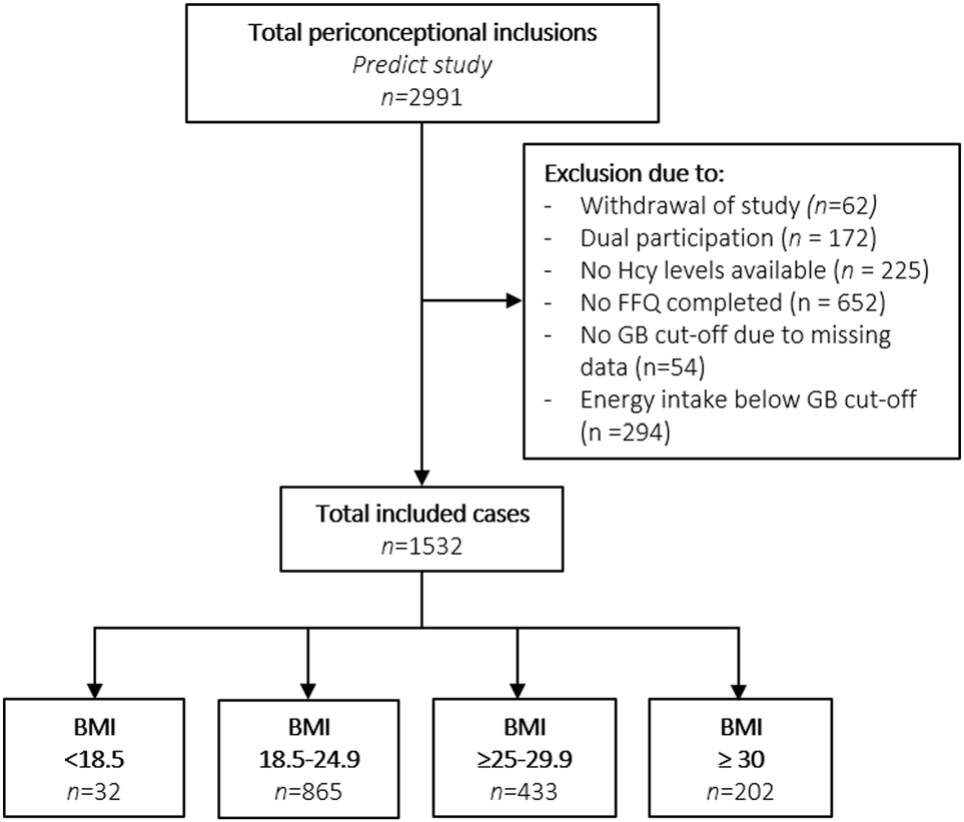
Flowchart of study population selection

The data used in this study were collected between January 2010 and December 2021 from the Rotterdam Periconceptional Cohort (Predict study), an ongoing prospective tertiary hospital-based cohort study at the Erasmus MC, Rotterdam, the Netherlands, and included 2991 participants. The detailed description of the recruitment process and used sampling techniques can be found in the study protocol papers of the Predict study [19, 20]. General inclusion criteria comprise women who are contemplating pregnancy or are less than 10 weeks pregnant with a singleton, have a minimum age of 18 years, and receive regular care at the Department of Reproductive Medicine or Obstetrics and Gynaecology at the Erasmus MC, Rotterdam, the Netherlands. We excluded participants who withdrew from the study (*n* = 62), who participated more than once (*n* = 172), if no tHcy concentrations were available (*n* = 225), with uncompleted general questionnaires (*n* = 652), with missing data for calculating Goldberg cut-offs (*n* = 54), and with unreliable intake data (*n* = 294). This resulted in a total of 1532 included participants for analysis, see the flowchart for the study population selection (Fig. 2). Participants were included with a follow up period of 1 year when included preconceptionally and up until 1 year after delivery when included in pregnancy. At study entry, blood samples were taken, anthropometric measurements were performed, and two questionnaires were filled out by the participants. Ethical approval for this study was obtained from the Medical Ethical and Institutional Review Board of the Erasmus MC, Rotterdam, the Netherlands (MEC-2004-277). All participants provided written consent prior to participation. 2991 women were included in the Rotterdam Periconceptional Cohort.

### Data collection

#### General characteristics

Data on maternal characteristics were extracted from the structured general questionnaire. Social and demographic characteristics included age and BMI, periconceptional (14 weeks before until 10 weeks after conception) smoking status, geographical origin categorized as Western (European (excluding Turkey), North America, Oceania, Japan, and Indonesia), or non-Western (Turkey, Africa, Asian, and Latin America) according to their country of birth and the classification of the Dutch Central Bureau of Statistics (CBS) [21], level of education classified as low, intermediate, or high according to the CBS [22], folic acid supplement use (periconceptional, minimum of 0.4 mg/day), multivitamin supplement use (periconceptional), and the additional intake of vitamin B_12_ and folic acid from dietary sources (in micrograms). BMI is categorized according to the classification of the World Health Organization (WHO) into underweight (BMI < 18.5 kg/m^2^), normal weight (18.5–24.9 kg/m^2^), overweight (BMI 25.0–29.9 kg/m^2^), and obesity (BMI ≥ 30 kg/m^2^).

#### Food Frequency Questionnaire and Classification of UPF

The Food Frequency Questionnaire (FFQ) is a semi-quantitative dietary assessment tool, designed and analyzed (using The Dutch Food Composition Database (NEVO)) by the Department of Human Nutrition, Wageningen University, the Netherlands [23]. It consists of an extensive list of foods and beverages with response categories to indicate the usual frequency of consumption, portion size, and preparation method in the participants’ diet during the past four weeks. The FFQ was filled out once by each participant, prior to the study intake. Participants included during pregnancy completed the questionnaires before a mean of 57 (SD ± 10) days of gestational age. For each participant, the total daily energy intake and the (micro)nutrient values of the separate food and beverage items were provided. Using dietary intake and 24-h recalls, the total energy and macronutrient intake reported by the FFQ were validated, including during pregnancy [24, 25]. The method of triads was used to validate specific micronutrient intakes, including folate and vitamin B_12_ [25, 26].

All the separate food and beverage items in the FFQ were classified into one of the NOVA groups 1–4: ‘unprocessed or minimally processed foods,’ ‘processed culinary ingredients,’ ‘processed foods,’ or ‘ultra-processed foods,’ respectively [27]. This classification was determined independently by three researchers among which nutritional expertise was present, using the nutritional values available from the (online) food labels (Supplemental Table 1). Discrepancies concerning the classification of any item were resolved through consensus in discussion.

#### Energy intake derived from ultra-processed foods

The percentage of energy intake derived from ultra-processed foods (PEI-UPF) was calculated for each participant by dividing the daily intake of UPF (in kilojoules) by the total reported energy intake (REI) (in kilojoules). To ensure the exclusion of participants with unrealistic reported intake, we used the Goldberg cut-off method [28]. The plausibility and validation of the reported total energy intake was based on the expected energy intake (EEI), which was calculated per participant, using the basal metabolic rate (BMR) and the physical activity level (PAL). In this study, a standard value for PAL of 1.55 was used, equal to the WHO value for ‘light’ activity [29]. The BMR was calculated using the Schofield equations, based on the sex, age, weight, and height of each individual participant. Next, the EEI per specific participant is calculated, with the corresponding 95% confidence intervals, these interval values are called the Goldberg cut-offs. Participants are included for analysis if their REI (calculated from the FFQ) falls within the Goldberg cut-off values of the EEI because their intake is considered plausible. Participants following an energy-restricted diet were manually checked by the researcher for plausible REI before being included.

#### Homocysteine levels

Blood samples were collected only at the study intake, preconceptional or before 10 weeks of gestational age (mean 57 (± 10 SD) days). Blood samples were collected in ethylenediaminetetraacetic acid (EDTA) tubes. In order to prevent artificial increases of tHcy concentrations, the EDTA tubes were carried on ice, and centrifugation of the plasma samples was performed within 10 min after sampling [30]. Total plasma homocysteine levels were measured by using Waters Quattro Premie liquid chromatography-tandem mass spectrometry system (Waters, Milford, Massachusetts, United States), with an interassay coefficient of variation of < 5.5%. The normal concentration of tHcy varies between 4.6 and 15 µmol/L, and increased homocysteine levels are defined as > 15 µmol/L [31].

### Statistical analyses

#### Selection of covariates

A directed acyclic graph (DAG) was composed to select a minimal sufficient adjustment set of variables (MSAS) and identify the direct effect of the UPF intake on maternal tHcy (Supplemental Fig. 1). The DAG was built by including all selected covariates that may affect the exposure (PEI-UPF) and the outcome (tHcy); afterward, all common causal pathways of any pair of variables already in the DAG were included. Variables in the MSAS blocked all non-causal and visualized the causal pathways between UPF intake and tHcy. Included in MSAS are age, BMI, smoking status, folic acid/multivitamin supplement use, and dietary folate/vitamin B_12_ intake.

#### Statistical tests

Baseline characteristics are shown for the total population and PEI-UPF quartiles. For continuous variables with parametric data, ANOVA was used, for non-parametric data the Kruskal–Wallis test, and for categorical data the Chi-square test. In order to assess the association between PEI-UPF and tHcy, univariable and multivariable linear regression analyses were conducted. The principal assumptions (linearity, independency, homoscedasticity, normality, and multi-collinearity) were tested and met [32].

Based on our DAG (Supplemental Fig. 1), the dietary intake of folate, vitamin B_12_, and BMI were tested for potential mediation in the association between PEI-UPF and tHcy, using model 4 of Hayes PROCESS macro v4.1. None of these variables were found to be significant mediators. An interaction analysis was performed in order to assess if the association between PEI-UPF and tHcy is moderated by the selected covariates. Therefore, interaction terms between the exposure (PEI-UPF) and the selected MSAS were created. Based on the literature and the argument that it is preferable to include additional spurious interactions in the analysis, rather than mistakenly overlook a ‘true’ interaction, we raised the type I error for the included interaction terms in the models with a *p*-value below 0.20. Requirements for sample size power were met for this purpose [33].

Four models were constructed: the first consisted of a univariable linear regression model between PEI-UPF and tHcy. The second multivariable model was adjusted for the MSAS as selected in the DAG (age, BMI, smoking status, folic acid/multivitamin supplement use, dietary folate/vitamin B_12_ intake), and the third model additionally included all the interaction terms as described before. The final and optimal model (model 4) was adjusted for the MSAS and the selected significant interaction terms.

In addition, considering our hypothesis that women with overweight and obesity consume higher amounts of UPF, and for illustrative purposes, we created subgroups based on the stratification of BMI. Analyses were performed in the multivariable models and were adjusted for the interaction terms and the MSAS, excluding BMI. All data analyses were performed using SPSS version 28.1. A two-sided *p*-value of < 0.05 was considered statistically significant.

## Results

### Study population and baseline characteristics

In total, 1532 participants were enrolled for analysis, of which 32 participants with underweight, 865 participants with normal weight, 433 with overweight, and 202 with obesity. The flowchart of the study population is shown in Fig. 2.

In Table 1 the baseline characteristics of the total included participants and stratification according to quartiles of the determinant PEI-UPF are shown. At study entry, the mean age of the participants was 32.5 (± 4.5) with a median BMI of 24.1 kg/m^2^ (21.8–27.2). The majority of the participants were Western (88.0%), highly educated (59.0%), and did not smoke tobacco (85.3%) during the periconceptional period. Almost the entire population (98.3%) used folic acid supplements ≥ 8 weeks prior to conception, whereas 71.5% used a multivitamin supplement. The median tHcy was 6.3 (5.3–7.3) µmol/L and the median total energy intake was 7881 (6733–9379) kJ per day. The PEI-UPF ranged from 16 to 90%, with a mean PEI-UPF of 48%. Significant differences in baseline characteristics were seen when stratified for the PEI-UPF quartiles. Women with an increased PEI-UPF are younger and have a higher BMI. Furthermore, people are lower educated, more often smokers, and levels of tHcy and total intake of kJ per day were higher. No differences were seen between the PEI-UPF quartiles and the geographical origin or supplement intake.

**Table 1:** Baseline characteristics of the study population (*n* = 1532) and stratification for PEI-UPF quartiles ^a^

	Total participants *n* = 1532	Q1 16.37–40.65% *n* = 383	Q2 40.66–48.46% *n* = 383	Q3 48.47–56.44% *n* = 383	Q4 56.45–89.74% *n* = 383	*P* value
Age (years)	32.5 [± 4.5]	33.47 [± 4.3]	32.7 [± 4.3]	32.2 [± 4.6]	31.5 [± 4.6]	** < 0.001**
BMI (kg/m^2^)	24.1 [21.8–27.2]	23.5 [21.5–26.3]	23.7 [21.5–26.5]	24.7 [22.1–27.8]	24.8 [22.1–28.2]	** < 0.001**
Geographical origin						0.069
Western	1306 (88.0)	319 (85.1)	331 (90.2)	324 (86.6)	332 (90.2)	
Non-western	178 (12.0)	56 (14.9)	36 (9.8)	50 (13.4)	36 (9.8)	
* Missing*	*48*	*8*	*16*	*9*	*15*	
Educational level						** < 0.001**
Low	112 (7.6)	15 (4.0)	17 (4.7)	26 (6.9)	54 (14.6)	
Middle	496 (33.4)	81 (21.7)	117 (32.1)	154 (41.1)	144 (39.0)	
High	875 (59.0)	278 (74.3)	231 (63.3)	195 (52.0)	171 (46.3)	
* Missing*	*49*	*9*	*18*	*8*	*14*	
Smoking						** < 0.001**
Yes	217 (14.7)	24 (6.5)	46 (12.6)	68 (18.2)	79 (21.5)	
No	1257 (85.3)	345 (93.5)	318 (87.4)	305 (81.8)	289 (78.5)	
* Missing*	*58*	*14*	*19*	*10*	*15*	
Folic acid supplements						0.079
Yes	1458 (98.3)	370 (98.7)	363 (99.5)	363 (97.1)	362 (98.1)	
No	25 (1.7)	5 (1.3)	2 (0.5)	11 (2.9)	7 (1.9)	
* Missing*	*49*	*8*	*18*	*9*	*14*	
Multivitamin supplements						0.052
Yes	1047 (71.5)	283 (75.9)	261 (71.5)	269 (71.7)	246 (66.7)	
No	423 (28.5)	90 (24.1)	104 (28.5)	106 (28.3)	123 (33.3)	
* Missing*	*50*	*10*	*18*	*8*	*14*	
tHcy (µmol/L)	6.3 [5.3–7.3]	6.0 [5.2–6.9]	6.2 [5.3–7.3]	6.2 [5.2–7.3]	6.7 [5.6–7.9]	** < 0.001**
Total intake (kJ/day)	7880 [6785–9464]	7541 [6421–8743]	7681 [6672–9103]	7984 [6708–9391]	8583 [7188–10216]	** < 0.001**

^a^Q1 (used as reference group) represents participants with the lowest PEI-UPF (16.37–40.65% of their total intake consists of ultra-processed foods), whereas Q4 includes participants with the highest PEI-UPF (56.45–89.74%). Values shown are mean (± SD) for age, median (IQR) for BMI, tHcy, and total intake. Geographical origin, educational level, smoking, and supplement use are presented as counts and percentages, *n*(%). *P* values are calculated using ANOVA for continuous variables, the Kruskal–Wallis test for two binary variables, or the Chi-square test for categorical variables

### Effect modification analysis

Possible effect modification between the exposure and included covariates was assessed by performing an interaction analysis. In Supplemental Table 2 the created and tested interaction terms in the multiple linear regression analyses are shown. Age, BMI, and dietary intake of Vitamin B_12_ proved to be significant moderators in the association between UPF intake and tHcy levels (resp. *p* = 0.03, *p* = 0.13, and *p* = 0.16). All three interaction terms were included in the final model.

### Primary analyses: regression models of PEI-UPF and tHcy levels

Women included in Q4 of PEI-UPF showed higher tHcy and higher total daily kJ intake (Table 1). Statistically significant associations were observed between PEI-UPF and tHcy in the total periconceptional population. Simple linear regression (basic model) showed a significant positive association between PEI-UPF and tHcy (*β* = 0.22, 95% CI 0.12–0.32). In other words, for every 10% increase in PEI-UPF, tHcy increased by 0.22 µmol/L. The model adjusted for the MSAS showed a smaller effect estimate (*β* = 0.19, 95% CI 0.08–0.30), while the explained variance was increased (Table 3). Subsequently, the interaction terms were additionally included as described before. For each interaction term presented, the other covariates in the interaction analysis are kept at mean value. Dietary vitamin B_12_ intake, age, and BMI were found to be moderators in the association between PEI-UPF and tHcy. In participants with high dietary vitamin B_12_ intake, low age and BMI, an association between PEI-UPF and tHcy is found, however, not within participants with a low dietary intake of vitamin B_12_, or high age or BMI. As a result of the effect moderation, the effect estimate cannot be interpreted as an increase in tHcy per determined increased value of PEI-UPF. To better understand the nature of the moderation effects, simple slope analyses for the significant interaction terms in the final model are presented ().

### Secondary analyses: stratified by BMI group

The BMI proved to be a significant moderator in the relation between UPF intake and tHcy. Consistent with these findings and our hypothesis that women with overweight or obesity consume more UPF, we decided to perform secondary analysis according to the BMI group, in order to better understand the effect of the BMI.

#### Study characteristics stratified for BMI

Statistically significant differences were observed between the BMI groups for all characteristics except age and dietary vitamin B_12_ intake (Table 2). Due to the small number of participants in the underweight group (*n* = 32), we excluded them from further analyses stratified by BMI. Participants with overweight or obesity smoked more often tobacco and used less folic acid and/or multivitamin supplements compared to participants with a normal weight. tHcy was increased in the population with overweight and obesity, compared to the population with a normal weight. The contribution of UPF to the total intake was significantly higher in participants with overweight and obesity compared to participants with a normal weight. The intake of folate derived from the diet is significantly lower in the group with overweight, compared to the group with normal weight and obesity, where no differences are observed in the dietary intake of vitamin B_12_.

**Table 2:** Characteristics of study population according to BMI stratification

	BMI < 18.5 kg/m^2^ *n* = 32	BMI 18.5– < 25 kg/m^2^ *n* = 865	BMI 25– < 30 kg/m^2^ *n* = 433	BMI ≥ 30 kg/m^2^ *n* = 202	***P* value**
Age (years)	30.7 [± 3.9]	32.6 [± 4.4]	32.4 [± 4.5]	32.1 [± 4.8]	0.324
Smoking					**0.020**
Yes	3 (9.4)	106 (12.7)	71 (17.3)	37 (19.1)	
No	29 (90.6)	731 (87.3)	340 (82.7)	157 (80.9)	
* Missing*	*0*	*28*	*22*	*8*	
Geographical origin					** < 0.001**
Western	27 (84.4)	766 (90.7)	354 (86.3)	158(80.7)	
Non-western	5 (15.6)	79 (9.3)	56 (13.7)	38 (19.3)	
* Missing*	*0*	*20*	*23*	*5*	
Educational level					** < 0.001**
Low	3 (9.4)	41 (4.9)	39 (9.5)	29 (14.9)	
Middle	6 (18.8)	245 (29.0)	152 (36.9)	93 (47.7)	
High	23 (71.9)	558 (66.1)	221 (53.6)	73 (37.4)	
* Missing*	*0*	*21*	*21*	*7*	
Folic acid supplements					**0.007**
Yes	32 (100.0)	836 (99.1)	403 (97.8)	187 (95.9)	
No	0	8 (0.9)	9 (2.2)	8 (4.1)	
* Missing*	*0*	*21*	*21*	*7*	
Multivitamin supplements					** < 0.001**
Yes	26 (81.3)	653 (75.3)	261 (63.3)	137 (70.3)	
No	6 (18.7)	208 (24.7)	151 (36.7)	58 (29.7)	
* Missing*	*0*	*21*	*21*	*7*	
Folate diet (mg)	0.25 [0.18–0.30]	0.26 [0.20–0.32]	0.23 [0.19–0.29]	0.26 [0.20–0.31]	**0.002**
Vitamin B_12_ diet (µg)	3.59 [2.57–5.30]	3.61 [2.77–4.86]	3.64 [2.83–4.92]	3.50 [2.84–4.40]	0.562
Total daily intake (kJ)	7794 [6332–9969]	7862 [6671–9235]	7809 [6639–9499]	8135 [7122–9613]	**0.014**
Total intake UPF (kJ)	3948 [2968–5038]	3666 [2811–4721]	3807 [2961–4955]	4216 [3327–5258]	** < 0.001**
PEI-UPF (%)	49.6 [41.9–56.2]	46.8 [39.6–54.8]	50.3 [41.7–57.5]	51.3 [43.8–58.5]	** < 0.001**
tHcy (µmol/L)	6.7 [5.9–9.1]	6.1 [5.3–7.2]	6.3 [5.4–7.4]	6.6 [5.4–7.5]	**0.038**

Values shown are mean (± SD) for age, median (IQR) for dietary folate, vitamin B_12_, total daily, total UPF intake, PEI-UPF, and tHcy. Smoking, geographical origin, educational level, and supplement use are presented as counts and percentages, *n*(%). *P* values are calculated using ANOVA for continuous variables, the Kruskal–Wallis test for two binary variables, or the Chi-square test for categorical variables

#### Regression analysis: PEI-UPF and tHcy levels

**Fig. 3:**
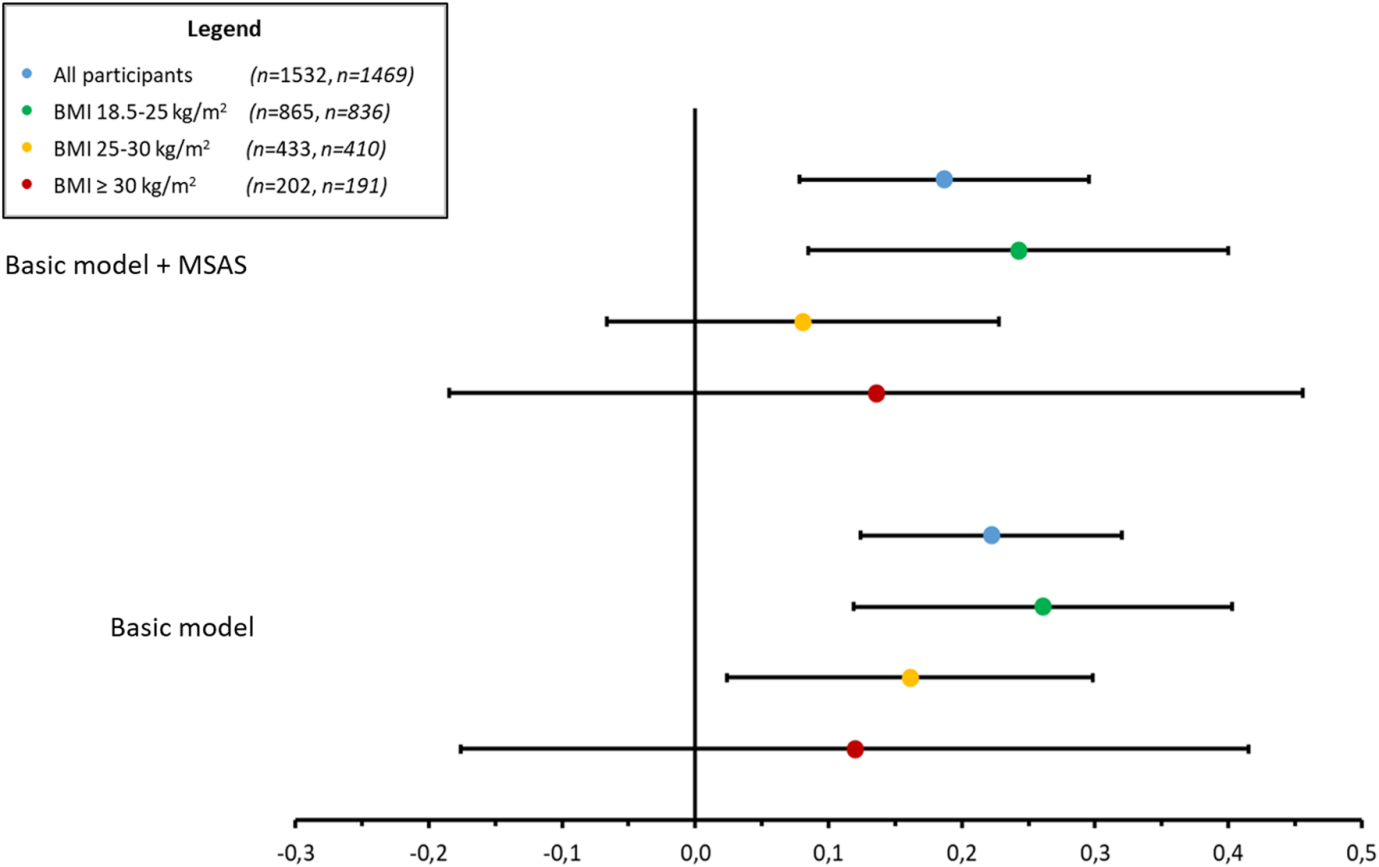
Forest plot of effect estimates of the associations between ultra-processed foods intake and tHcy, arranged according to the depicted models and BMI classification. Basic model: unadjusted. Basic model + Covariates: Adjusted for MSAS (minimal sufficient adjustment set) covariates identified in the DAG (directed acyclic graph): age, BMI, smoking status, folate and vitamin B_12_ dietary intake, folic acid, and multivitamin supplement use. *In the analysis stratified for BMI: no adjustments were made for BMI and the interaction term PEI-UPF*BMI was excluded

Significant positive associations between PEI-UPF and tHcy were found in all models in the population with normal weight. Both the basic and adjusted models for the MSAS showed a higher effect estimate and increased explained variance compared to the total population (Table 3 and Fig. 3). The models adjusted for the interaction terms showed large effect estimates for the association in PEI-UPF and tHcy; however, it should be kept in mind that these effect estimates change according to the included interaction terms and therefore cannot be interpreted individually. In the population with overweight a positive association between PEI-UPF and tHcy was observed. However, after adjustment for the MSAS, the association remained no longer significant and turned even negative in the model adjusted for the interaction terms. In order to assess the observed decrease and inverse association, an additional sequential regression analysis was performed (Supplemental Table 3). Dietary intake of folate appeared to be responsible for the reduced effect estimate. The inverse effect estimate in the third model is observed after adding the interaction term PEI-UPF*folic acid supplement use. In the participants with obesity, no significant associations were observed between PEI-UPF and tHcy in any of the models.

**Table 3:** Associations between periconceptional ultra-processed foods intake and tHcy in the total population and stratified for BMI groups

	*β*	95% CI	*p* value	*n*	Adjusted *R* square
All participants					
Basic model^a^	0.22	0.12–0.32	< 0.001	1532	0.012
Basic model + MSAS^b^	0.19	0.08–0.30	< 0.001	1469	0.022
Basic model + MSAS + all IT^c^	1.27	0.05–2.49	0.04	1469	0.024
Basic model + MSAS + selected IT^d^	1.31	0.38–2.23	0.01	1469	0.026
BMI 18.5–24.9 kg/m^2*^					
Basic model^a^	0.26	0.12–0.40	< 0.001	865	0.014
Basic model + MSAS^b^	0.24	0.09–0.40	0.003	836	0.018
Basic model + MSAS + all IT^c^	3.30	0.75–5.85	0.01	836	0.031
Basic model + MSAS + selected IT^d^	1.07	0.06–2.07	0.04	836	0.030
BMI 25.0–29.9 kg/m^2*^					
Basic model^a^	0.16	0.02–0.23	0.02	433	0.010
Basic model + MSAS^b^	0.08	− 0.07–0.23	0.28	410	0.059
Basic model + MSAS + all IT^c^	− 0.43	− 1.72–0.85	0.51	410	0.057
Basic model + MSAS + selected IT^d^	0.06	− 0.95–1.07	0.90	410	0.054
BMI ≥ 30 kg/m^2*^					
Basic model^a^	0.12	− 0.18–0.42	0.43	202	− 0.002
Basic model + MSAS^b^	0.14	− 0.19–0.46	0.40	191	0.003
Basic model + MSAS + all IT^c^	1.49	− 1.17–4.16	0.27	191	0.029
Basic model + MSAS + selected IT^d^	1.70	− 0.52–3.92	0.13	191	0.006

^a^Basic model: unadjusted. ^b^Basic model + MSAS (minimal sufficient adjustment set of variables): Adjusted for MSAS identified in the DAG (directed acyclic graph): age, BMI, smoking status, folate and vitamin B_12_ dietary intake, folic acid and multivitamin supplement use. ^c^Basic model + MSAS + all IT (interaction terms): adjusted for MSAS and all interaction terms. ^d^Basic model + MSAS + selected IT: Adjusted for MSAS and significant interaction terms. ^*^In the analysis stratified for BMI: no adjustments were made for BMI and the interaction term PEI-UPF*BMI was excluded

### Tertiary analysis: including under reporters

Additional analyses were conducted to provide more information about the effects of excluding under reporters from our dataset. Excluded participants, based on the Goldberg cut-offs (*n* = 294), reported 40% less total intake (kJ/day) and lower PEI-UPF compared to included participants. Furthermore, they were significantly younger, had an increased BMI, were more often of non-Western geographical origin, and were less educated compared to included participants (Supplemental Table 4).

The regression models in the total population and normal weight population showed overall lower effect estimates, lower explained variance, and wider confidence intervals when including the under reporters (Supplemental Table 5). Furthermore, the models including interaction terms were no longer found to be significant. In the overweight participants, significant associations were found in the basic and adjusted model, but not in the models including interaction terms. No significant differences were found in the associations of the participants with obesity when including underreporters.

## Discussion

### Summary of findings

This study confirms the hypothesis that increased PEI-UPF is associated with increased tHcy in our total study population during the periconceptional period. The association is moderated by dietary intake of vitamin B_12_, age, and BMI. Stratification of the population according to the BMI classification shows comparable findings in the participants with normal weight and overweight. However, the associations no longer remain significant after adjustment for multiple covariates in the participants with overweight. Analyses of the participants with obesity showed no significant associations between PEI-UPF and tHcy.

### Interpretation of findings and comparison with other studies

So far, limited studies investigated UPF intake in relation to homocysteine or 1-CM in a reproductive population, but associations between the consumption of ultra-processed foods and a reduction in the quality of diet in pregnant women are found [34, 35]. Indeed, early in pregnancy, it was found that high maternal UPF intake is associated with impaired first-trimester growth; however, when adjusting for B vitamins the association became less strong and non-significant, suggesting the found effect is based on (lack of) nutrient intake, metabolization, and/or absorption [36].

It is remarkable that PEI-UPF only has a significant effect on tHcy in the total and normal weight population, especially since PEI-UPF is higher in the population with overweight and obesity. Several reasons may underlie this observation. First, there are pathophysiological changes in people with obesity that contribute to a low-grade inflammatory state. The intake of excessive amounts of calories contributes to the dysfunctional lipid storage in adipocytes, resulting in adipose tissue dysfunction, which is observed in people with obesity and characterized by a loss of homeostatic functions, infiltration of immune cells, and increased production of pro-inflammatory cytokines [37]. The increased amount of immune cells in adipose tissue produces chemokines and reactive oxygen species (ROS) which may affect adjacent tissues and organs resulting in an oxidative stress state [38]. Regeneration and proliferation of the damaged tissue cells are supported by the 1-CM moieties, but deficiencies of B vitamins and folate in the relative status of malnutrition in women with overweight/obesity dysregulate the 1-CM, leading to HHcy [39]. This implies that the chronic inflammatory state and B vitamin deficiencies could potentially overrule the effect of PEI-UPF on tHcy and explains why the effect is only observed in participants with normal weight.

Second, the association between PEI-UPF and tHcy proved to be significantly moderated by age, BMI, and dietary vitamin B_12_ intake. In participants with high BMI or age, the association between PEI-UPF and tHcy was not observed, as compared to younger participants with normal BMI. This may suggest that these biological factors are more important in the association with tHcy than the PEI-UPF.

Another important result is the strong decrease in effect estimate in the overweight population due to dietary folate intake. Suggesting that a balanced diet, including folate-rich foods, seems to counteract the adverse effects of a diet high in UPF. Literature shows that people consuming UPF in a varied diet, well-balanced in terms of calories and nutrients, are not experiencing the same impact on tHcy levels as when consumption of UPF in a high-calorie diet leads to a reduction or substitution of nutritional foods [40]. After all, food consists of complex combinations of nutrients and other components that act synergistically within and between diets and lifestyles.

So far, limited research has investigated the effects of UPF independent of their nutrient content. Some studies reported associations between the intake of UPF, weight gain, obesity, and diseases including, diabetes, hypertension, and gastrointestinal disorders, which persisted after adjusting for total intake and dietary content, including saturated fat, added sugar, and sodium [41–43]. Suggesting there may be other factors than the nutritional content of the diet contributing to the adverse health outcomes. Therefore, another important question remains whether the observed associations between the PEI-UPF and tHcy are a manifestation of a poor nutritional diet, a consequence of the actual food processing, or a combination. Other possible mechanisms, besides a low nutritional content, are changes in food matrix and texture, which make UPF easier to digest and absorb by the body. Additionally, contaminants are created during food processes, such as trans fats and advanced glycation end-products, which are detrimental to fat metabolism and disrupt glycemic homeostasis [15].

Besides the impact on nutrient deficiencies, a diet high in UPF also negatively influences the functionality and diversity of the gut microbiota [44]. The gut microbiota plays an important role in breaking down food components, which cannot be digested by the present human gut enzymes. There is a reciprocal interaction, with the gut bacteria processing B vitamins of the 1-CM, while the micronutrients of the 1-CM are essential in the maintenance of gut microbiota homeostasis [18]. Dysbiosis of gut microbiota during pregnancy is associated with offspring predisposition to metabolic and abnormalities in neurodevelopment [18]. Gradually, more literature is emerging on *in-utero* or transplacental colonization of offspring, apart from the vertical transfer of gut microbiota from the mother to child during delivery [45]. Pregnant mice on a high-fat diet showed alterations in their gut microbiota, and their offspring likewise showed significantly lower overall bacterial abundance, a higher *Firmicutes-*to-*Bacteroidetes* ratio, higher percentages of body fat, and altered immune development compared to the mice following a normal diet [46]. On the other side of the reciprocal interaction, considering the effects on the 1-CM, gut dysbiosis leads to a decrease in *Bacteroidetes* bacteria and their production of B vitamins, and an increase in *Firmicutes* bacteria*,* responsible for energy harvest [18]. As a consequence, folate and vitamin B12 are synthesized to a lesser extent, leading to relative vitamin deficiencies, derangements of the 1-CM, and higher tHcy levels. Subsequently, the derangements of the 1-CM additionally contribute to the already present low-grade systemic inflammation in the population with overweight or obesity, which completes the vicious circle [47].

### Strengths and limitations

To our best knowledge, this is the first study to investigate the associations between the intake of UPF and tHcy in the periconceptional population. A strength of this study is the combination of sample size and extensive data collection on maternal characteristics. These characteristics provide the opportunity to adjust for potential confounding, important to approximate the estimates of the associations between UPF intake and tHcy. Furthermore, our population showed a variable distribution in PEI-UPF (16–90%), by which the effect of UPF on tHcy can be assessed across the full exposure range. Ideally, an additional analysis was performed, investigating whether different levels of tHcy are found when comparing high and low PEI-UPF in participants with overweight and obesity. Unfortunately, this was not feasible due to a lack of sample size in our population with overweight/obesity. Furthermore, we selected our population from a hospital-based high-risk cohort study; therefore, the results of this study are not generalizable to the general population.

Against expectations, the reported median daily energy intake in the total and normal weight population (resp. 7880 kJ and 7862 kJ) in our study is not substantially lower than in women with obesity (8135 kJ) and even higher than in the group with overweight (7809 kJ). This variance of around 200–300 kJ (or 60–75 kcal) does not align with the described excessive intake of kcal or kJ in people with obesity causing weight gain [13]. One explanation might be that people with overweight are more likely to follow a weight-reduction diet. However, in our study, this fact was known to every participant and accounted for. Recall bias, more common in lower educated and women with overweight and obesity, may primarily lead to underreporting of the total intake but also to specific categories of products, rich in fat and/or carbohydrates [48, 49]. Due to the plausible underreporting, the effects we find are likely to be underestimated. Despite the fact that under reporters were excluded by using the Goldberg cut-offs, it is only certain that participants were excluded with an unrealistic total energy intake. A further limitation to address is the use of the semi-quantitative FFQ in our study. This questionnaire is not explicitly designed and structured to measure UPF intake, and therefore, the input from the researchers and dieticians was required to categorize the foods according to the Nova classification, possibly affecting precision [50]. Another limitation is the measurement of tHcy in preconceptional and first-trimester women, as we know there is a decrease in tHcy over the course of pregnancy. However, in literature, the decrease is primarily explained by the use of folic acid. In our population, the use of folic acid is comparable between preconceptional and pregnant inclusions, due to the fact that our preconceptional population is included at the outpatient clinic of Reproductive Medicine, where they are strongly advised to use folic acid. Lastly, the possibility of residual confounding in the association between PEI-UPF and tHcy. Although we have an extensive set of covariates at our disposal, still certain covariates that are not measured may affect the association. These factors include medication use, components in multiple micronutrient supplements or from dietary intake (including vitamin B_6_), and inherited diseases that might increase tHcy.

### Implications and recommendations for future research

The question arises whether B vitamin deficiencies are a direct consequence of obesity itself, the result of an imbalanced diet/reduced intake, or a combination of these aspects. Therefore, an important ensuing question is whether supplementation of B vitamins in synthetic form or via the diet can restore 1-CM, lower tHcy, and subsequently reduce ROS. In our (normal weight) population the association between PEI-UPF and tHcy remained significantly positive even after adjustment for dietary intake of folate and vitamin B_12_ and supplement intake as determinants of tHcy. Several studies investigated supplementation with either natural or synthetic folic acid or vitamin B complexes in relation to tHcy. They showed that supplementation significantly lowers tHcy, ROS, and anti-oxidant levels in participants with a normal weight [38, 51, 52]. Furthermore, a higher serum folate status was associated with lower BMI in patients with HHcy, but no studies investigated supplementation of vitamin B complexes in the population with overweight or obesity [53].

## Conclusion

This study showed that higher PEI-UPF is associated with higher tHcy in the high-risk periconceptional population. The association is moderated by periconceptional age, BMI, and dietary vitamin B_12_ intake. In the population with overweight or obesity, other systemic factors, including low-grade inflammatory state, may outweigh the contribution of PEI-UPF to increased tHcy. Focus on lifestyle, diet, and weight loss in the population with overweight or obesity is important, but there is also a window of opportunity to provide targeted advice on dietary quality and reduction of UPF consumption in the periconceptional population including women having a normal weight. Better knowledge regarding nutritional values of the diet may lead to a decrease in tHcy, restoration of the 1-CM and gut homeostasis, and eventually improve pregnancy, transgenerational, and long-term maternal health.

### Supplementary Information

Below is the link to the electronic supplementary material.

Supplementary file1 (DOCX 2238 KB)

## Data Availability

The data that support the findings of this study are available from the corresponding author upon reasonable request.
