# Supplementary material for: The intake of ultra-processed foods and homocysteine levels in women with(out) overweight and obesity: The Rotterdam Periconceptional Cohort
Source: Eur J Nutr. 2024 Feb 21;63(4):1257–69. doi: 10.1007/s00394-024-03334-w (PMC11139698; doi:10.1007/s00394-024-03334-w)
Supplement: Supplementary file 1 — Supplementary file1 (DOCX 2238 KB) [file 394_2024_3334_MOESM1_ESM.docx]

**Supplemental Material**

**Supplemental Fig 1.** Directed Acyclic Graph (DAG) for percentage of energy intake from ultra-processed foods (PEI-UPF) and total homocysteine plasma level (tHcy).
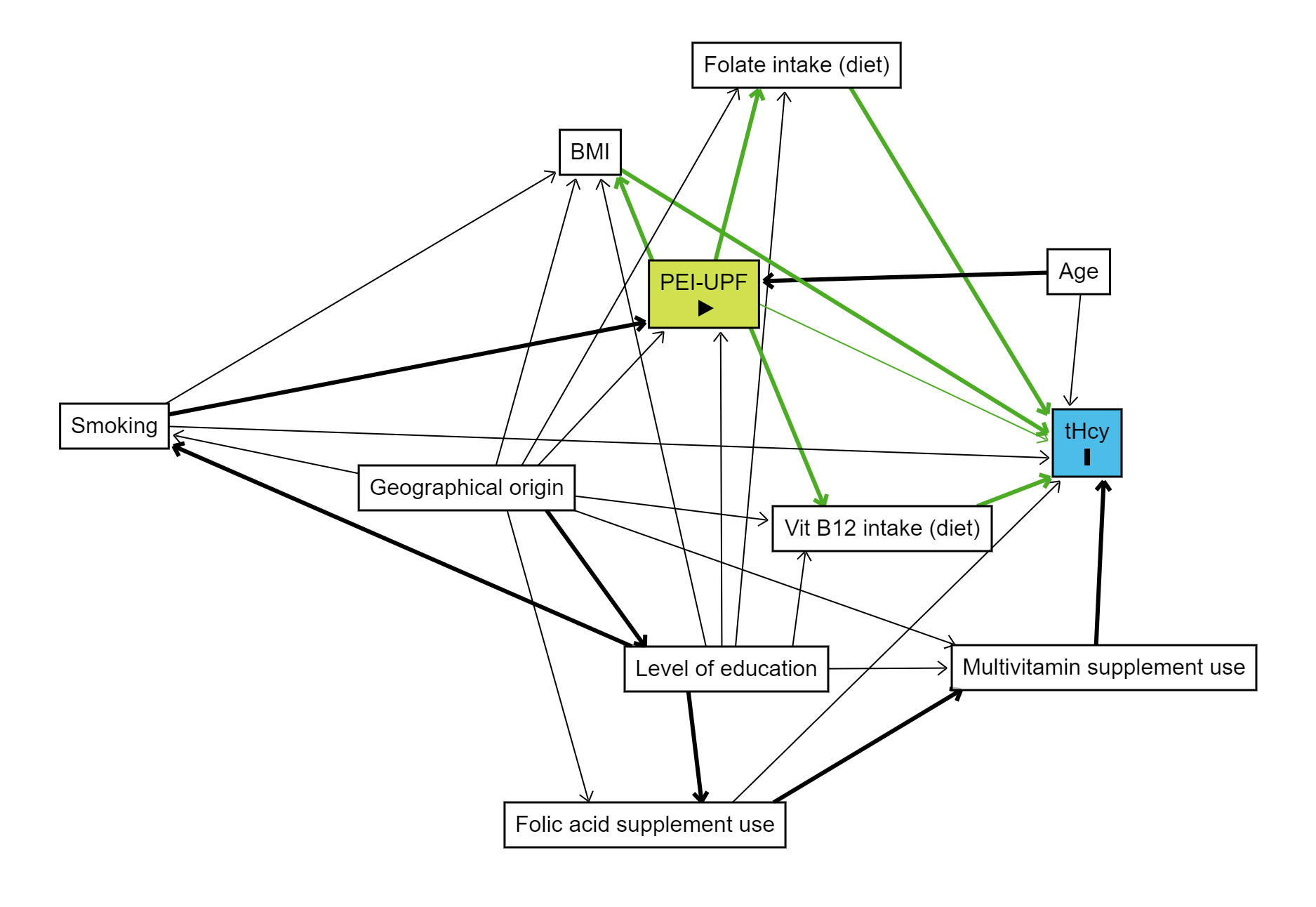


**Supplemental Figure 2.** Simple slopes for the included interaction terms UPF*Age (A), UPF*BMI (B) and UPF*Dietary vitamin B_12_ intake in the association between UPF intake en tHcy levels.


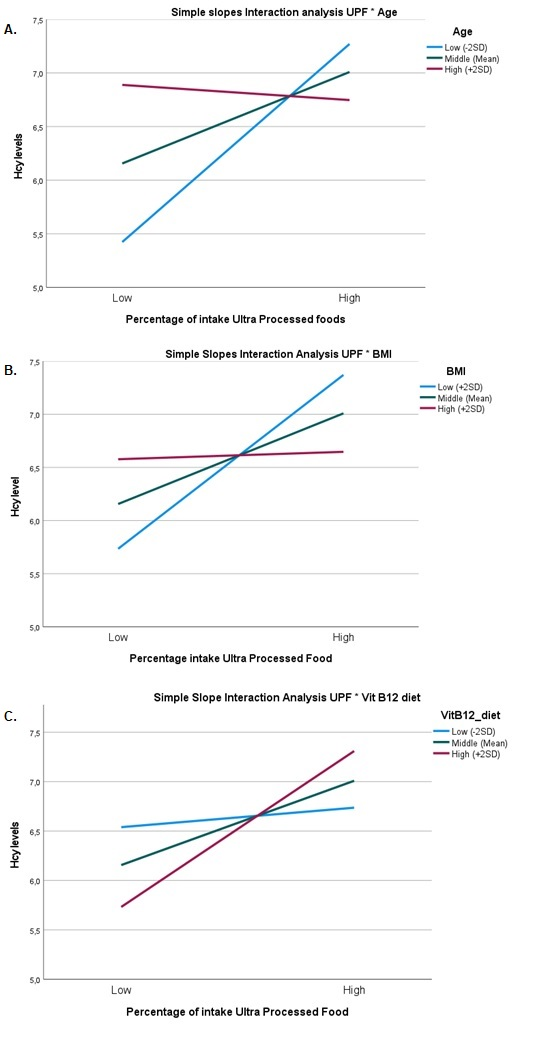

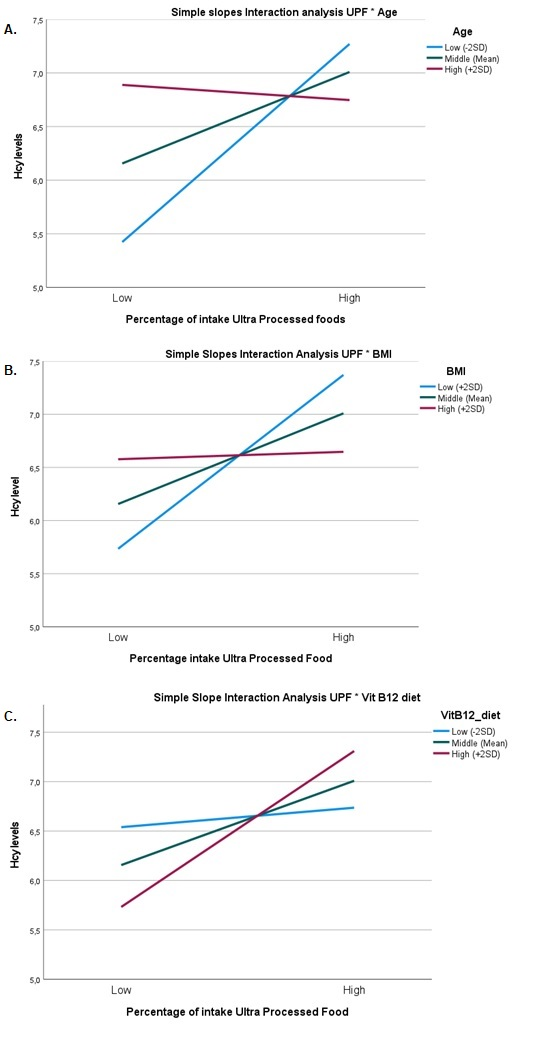


**Supplemental Table 1.** Classification of items reported in the FFQ according to responding NOVA group.

| **NOVA group 1** | | **NOVA group 2** | | **NOVA group 3** | | **NOVA group 4** | |
| --- | --- | --- | --- | --- | --- | --- | --- |
| **ITEM ID** | **ITEM** | **ITEM ID** | **ITEM** | **ITEM ID** | **ITEM** | **ITEM ID** | **ITEM** |
| 11 | boiled potatoes or mashed potatoes (also in stew) | 129 | Yeast extract or yeast paste | 26 | beer | 3 | fried or baked potatoes, potato slices, croquettes, rösti, etc. |
| 59 | coffee | 553 | Sugar | 37 | sherry, port, vermouth, etc | 7 | French fries (home-fried) |
| 62 | tea | 582 | buttercream | 39 | wine | 30 | mixed drinks (like breezer) |
| 76 | vegetable juice | 583 | semi-skimmed butter | 42 | low-alcohol or alcohol-free beer | 32 | spirits |
|  |  | 588 | Frying fat (solid) | 112 | brown bread | 44 | Drinking breakfast |
| 83 | orange juice | 591 | olive oil | 114 | multigrain bread | 49 | soft drink, fruit lemonade, sports drink or energy drink |
| 84 | apple juice | 592 | sunflower oil, soybean oil, salad oil, etc. | 116 | wholemeal bread | 98 | rusk, crispbread or crackers |
| 85 | other fruit juices | 593 | bacon fat or beef fat | 118 | white bread | 120 | raisin, currant or granola bread |
| 136 | eggs | 589 | liquid frying product | 304 | 20+ or 30+ cheese | 122 | rye bread |
| 141 | Citrus fruits | 796 | unknown type of cooking fat | 306 | 40+ or 48+ full-fat cheese | 126 | croissants |
| 146 | apple | 367 | diet coffee milk (linoleic acid-rich) | 313 | cream cheese or foreign cheese | 179 | large cookies or slices of cake |
| 148 | banana | 369 | halvamel or skimmed evaporated milk | 494 | pancakes | 184 | small cookies or sponge cake |
| 160 | Other fresh fruits | 374 | whole evaporated milk or coffee cream | 608 | Tomato sauces | 189 | nutritional cookies (liga, sultana, etc) or granola bars |
| 169 | dried fruits | 392 | creme fraiche or other prepared cream | 626 | smoked or steamed fish (e.g., salmon, mackerel) or sardines, canned herring | 194 | pastry or cake |
| 233 | whole wheat pasta or other pasta | 792 | unknown type of evaporated milk | 651 | minced meat (all types) | 207 | gingerbread |
| 235 | white pasta or other pasta |  |  | 679 | pork bratwurst or lettuce wing | 225 | muesli, cruesli |
| 238 | white rice |  |  | 770 | salted herring | 226 | high-fiber breakfast products such as All Bran |
| 240 | brown rice |  |  | 786 | other types of bread | 227 | cornflakes |
| 242 | grains such as bulgur, couscous, millet (no rice) |  |  | 788 | onbekend soort snijdbare kaas | 228 | other breakfast products |
| 247 | cauliflower and broccoli |  |  | 789 | unknown type of cuttable cheese | 293 | sandwichspread |
| 250 | onion |  |  |  |  | 296 | peanut butter or nut paste |
| 260 | cabbages (white, red, pointed, green, savoy, Chinese, kale, sauerkraut and Brussels sprouts) |  |  |  |  | 307 | cheese spread or dairy spread |
| 265 | other cooked or stir-fried vegetables |  |  |  |  | 346 | semi-skimmed chocolate milk |
| 276 | raw vegetables |  |  |  |  | 348 | skimmed chocolate milk |
| 337 | semi-skimmed milk |  |  |  |  | 349 | full-fat chocolate milk |
| 338 | buttermilk |  |  |  |  | 359 | ice cream or milk-based ice cream |
| 339 | skimmed milk |  |  |  |  | 361 | water ice or sorbet ice cream |
| 340 | nuts, nut mix, student oats |  |  |  |  | 372 | coffee creamer |
| 426 | seeds or kernels |  |  |  |  | 396 | whipped cream |
| 429 | legumes |  |  |  |  | 398 | custard or pudding |
| 452 | lean fish such as cod, plaice, tilapia, pangasius, trout, tuna, etc. |  |  |  |  | 412 | semi-skimmed (fruit) yogurt or (fruit) cottage cheese |
| 617 | Fatty fish such as salmon, mackerel, eel, pan herring |  |  |  |  | 415 | skimmed (fruit) yogurt or (fruit) cottage cheese |
| 623 | Prawns |  |  |  |  | 419 | full-fat (fruit) yogurt or (fruit) cottage cheese |
| 636 | mussels |  |  |  |  | 431 | chips or salts |
| 642 | other seafood |  |  |  |  | 439 | deep-fried savory snacks |
| 644 | chicken or other poultry |  |  |  |  | 441 | Non deep-fried savory snacks |
| 656 | other types of meat and game |  |  |  |  | 444 | bread salads with fish |
| 668 | liver |  |  |  |  | 446 | bread salads without fish |
| 670 | beef steak, beef tartare, beef fryer, roast beef |  |  |  |  | 497 | pizza |
| 675 | beef entrecote, beef sausage, beef knuckle steak, beef rib steak, marbled beef steak |  |  |  |  | 503 | oriental meal |
| 676 | bacon slices or bacon bits |  |  |  |  | 514 | soup with legumes |
| 678 | pork tenderloin, pork schnitzel, pork fricandeau, pork ham steak |  |  |  |  | 516 | soup without legumes |
| 680 | pork chops (shoulder, rib and haunch chops) |  |  |  |  | 519 | soy milk, soy drink or soy dessert |
| 681 | other pork |  |  |  |  | 527 | Quorn meat substitute |
| 682 | spinach |  |  |  |  | 529 | Valess meat substitute |
| 747 | green beans, string beans and broad beans |  |  |  |  | 531 | tofu/tempé/soya products |
| 749 | carrot |  |  |  |  | 533 | other meat substitutes |
| 751 | kiwi |  |  |  |  | 540 | bonbons |
| 759 | Strawberry’s |  |  |  |  | 544 | chocolates |
| 761 | Boiled liver |  |  |  |  | 548 | candy |
| 768 | unknown type of milk |  |  |  |  | 566 | other sweet spreads |
| 790 | unknown type of fish |  |  |  |  | 571 | halvarine or light margarine |
| 799 | unknown type of meat |  |  |  |  | 572 | diet halvarine or diet margarine light |
| 800 | Other beef meat |  |  |  |  | 573 | halvarine with plant sterols/stanols |
| 801 |  |  |  |  |  | 574 | low-fat halvarine product (25% fat or less) |
|  |  |  |  |  |  | 576 | margarine in tub |
|  |  |  |  |  |  | 577 | margarine in package |
|  |  |  |  |  |  | 578 | diet margarine |
|  |  |  |  |  |  | 579 | margarine with plant sterols/stanols |
|  |  |  |  |  |  | 585 | baking and frying product from a packet |
|  |  |  |  |  |  | 586 | cooking product from a bottle, liquid margarine |
|  |  |  |  |  |  | 597 | dressing or salad sauce |
|  |  |  |  |  |  | 598 | clear dressing without oil |
|  |  |  |  |  |  | 600 | mayonnaise |
|  |  |  |  |  |  | 601 | halvanaise, chipotle sauce or other non-red sauces |
|  |  |  |  |  |  | 606 | satay sauce |
|  |  |  |  |  |  | 610 | other hot sauces |
|  |  |  |  |  |  | 612 | jus |
|  |  |  |  |  |  | 619 | fish sticks |
|  |  |  |  |  |  | 621 | steak or kibble |
|  |  |  |  |  |  | 662 | hamburger |
|  |  |  |  |  |  | 666 | smoked sausage or frankfurters |
|  |  |  |  |  |  | 690 | smoked meat, fricandeau, roast beef, casser rib, chicken breast, chicken roast, filet americain |
|  |  |  |  |  |  | 692 | other meats |
|  |  |  |  |  |  | 694 | ham, such as back ham, shoulder ham, ham on the bone, raw ham |
|  |  |  |  |  |  | 700 | brown buns |
|  |  |  |  |  |  | 706 | liver sausage, spread sausage, pate, liver pate, liver cheese |
|  |  |  |  |  |  | 710 | multigrain buns |
|  |  |  |  |  |  | 712 | wholemeal buns |
|  |  |  |  |  |  | 714 | white rolls |
|  |  |  |  |  |  | 723 | currant bun or muesli bun |
|  |  |  |  |  |  | 726 | candy bars or chocolate candies |
|  |  |  |  |  |  | 738 | sausages such as sandwich sausage, cervelate sausage, salami, cooked sausage, roast minced meat |
|  |  |  |  |  |  | 765 | chocolate spread |
|  |  |  |  |  |  | 782 | savory pie |
|  |  |  |  |  |  | 784 | semi-skimmed drinking yogurt or dairy drink |
|  |  |  |  |  |  | 785 | skimmed drinking yogurt or dairy drink |
|  |  |  |  |  |  | 787 | unknown type of breakfast cereal |
|  |  |  |  |  |  | 789 | unknown type of spreadable cheese |
|  |  |  |  |  |  | 791 | unknown type of chocolate milk |
|  |  |  |  |  |  | 793 | unknown type of yogurt or cottage cheese |
|  |  |  |  |  |  | 794 | unknown type of meat substitute |
|  |  |  |  |  |  | 795 | unknown type of spread |
|  |  |  |  |  |  | 798 | unknown type of sauce |
|  |  |  |  |  |  | 802 | unknown type of cured meats |
|  |  |  |  |  |  | 803 | unknown type of buns/rolls |
|  |  |  |  |  |  | 804 | unknown type of drinking yogurt or dairy drink |
|  |  |  |  |  |  | 797 | unknown type of dressing |
|  |  |  |  |  |  | 79 | fruit drink, double drink or multifruit drink |
|  |  |  |  |  |  | 424 | peanuts or branuts |
|  |  |  |  |  |  | 505 | italian pasta meal, e.g. spaghetti, lasagna, etc. |
|  |  |  |  |  |  | 602 | tomato ketchup or other pre-packed red sauces |

**Supplemental Table 2.** Moderated multiple linear regression analysis testing the association between ultra-processed foods intake and homocysteine levels in the overall population

|  |  |  |  |
| --- | --- | --- | --- |
|  | **β** | **Standard Error** | **p-value** |
|  |  |  |  |
| PEI-UPF* | 1,269 | 0,624 | 0.042 |
| BMI * PEI-UPF | -0,019 | 0,013 | **0.131** |
| Age * PEI-UPF | -0,024 | 0,011 | **0.031** |
| Smoking status * PEI-UPF | -0,025 | 0,164 | 0.881 |
| Dietary Folate * PEI-UPF | 0,083 | 0,581 | 0.886 |
| Dietary Vitamin B_12_ * PEI-UPF | 0,036 | 0,025 | **0.155** |
| Folic acid supplement * PEI-UPF | 0,063 | 0,345 | 0.854 |
| Multivitamin supplement * PEI-UPF | -0,054 | 0,115 | 0.641 |

**Supplemental Table 3.** Sequential analysis of all interaction terms in the association between ultra-processed foods and homocysteine levels.

| Model | | Unstandardized Coefficients | | Sig. |
| --- | --- | --- | --- | --- |
|  |  | B | Std. Error |  |
| 1 | **(Constant)** | 6,320 | 0,972 | <0,001 |
|  | **PEI-UPF** | 0,081 | 0,075 | 0,279 |
|  | **Age** | 0,042 | 0,018 | 0,023 |
|  | **Smoking status** | 0,033 | 0,216 | 0,877 |
|  | **Folic acid supplement intake** | -0,336 | 0,555 | 0,546 |
|  | **Multivitamin supplement intake** | -0,705 | 0,171 | <0,001 |
|  | **Dietary Folate intake** | -2,433 | 1,045 | 0,020 |
|  | **Dietary Vitamin B_12_ intake** | -0,029 | 0,034 | 0,391 |
| 2 | **(Constant)** | 6,392 | 2,681 | 0,018 |
|  | **PEI-UPF** | 0,067 | 0,503 | 0,895 |
|  | **Age** | 0,040 | 0,077 | 0,608 |
|  | **Smoking status** | 0,033 | 0,216 | 0,878 |
|  | **Folic acid supplement intake** | -0,336 | 0,556 | 0,546 |
|  | **Multivitamin supplement intake** | -0,705 | 0,171 | <0,001 |
|  | **Dietary Folate intake** | -2,436 | 1,051 | 0,021 |
|  | **Dietary Vitamin B_12_ intake** | -0,029 | 0,034 | 0,393 |
|  | **PEI-UPF*Age** | 0,000 | 0,015 | 0,977 |
| 3 | **(Constant)** | 5,925 | 2,717 | 0,030 |
|  | **PEI-UPF** | 0,163 | 0,511 | 0,750 |
|  | **Age** | 0,049 | 0,078 | 0,526 |
|  | **Smoking status** | 1,050 | 0,984 | 0,287 |
|  | **Folic acid supplement intake** | -0,374 | 0,557 | 0,503 |
|  | **Multivitamin supplement intake** | -0,706 | 0,171 | <0,001 |
|  | **Dietary Folate intake** | -2,418 | 1,051 | 0,022 |
|  | **Dietary Vitamin B_12_ intake** | -0,028 | 0,034 | 0,409 |
|  | **PEI-UPF*Age** | -0,001 | 0,015 | 0,926 |
|  | **PEI-UPF*Smoking** | -0,197 | 0,186 | 0,291 |
| 4 | **(Constant)** | 5,859 | 2,799 | 0,037 |
|  | **PEI-UPF** | 0,175 | 0,526 | 0,740 |
|  | **Age** | 0,048 | 0,079 | 0,539 |
|  | **Smoking status** | 1,046 | 0,986 | 0,290 |
|  | **Folic acid supplement intake** | -0,373 | 0,558 | 0,504 |
|  | **Multivitamin supplement intake** | -0,706 | 0,172 | <0,001 |
|  | **Dietary Folate intake** | -2,035 | 3,999 | 0,611 |
|  | **Dietary Vitamin B_12_ intake** | -0,027 | 0,034 | 0,419 |
|  | **PEI-UPF*Age** | -0,001 | 0,015 | 0,939 |
|  | **PEI-UPF*Smoking** | -0,196 | 0,187 | 0,294 |
|  | **PEI-UPF*Dietary Folate** | -0,079 | 0,797 | 0,921 |
| 5 | **(Constant)** | 5,882 | 2,811 | 0,037 |
|  | **PEI-UPF** | 0,169 | 0,529 | 0,749 |
|  | **Age** | 0,048 | 0,079 | 0,538 |
|  | **Smoking status** | 1,045 | 0,987 | 0,291 |
|  | **Folic acid supplement intake** | -0,367 | 0,561 | 0,513 |
|  | **Multivitamin supplement intake** | -0,705 | 0,172 | <0,001 |
|  | **Dietary Folate intake** | -1,916 | 4,182 | 0,647 |
|  | **Dietary Vitamin B_12_ intake** | -0,042 | 0,151 | 0,781 |
|  | **PEI-UPF*Age** | -0,001 | 0,016 | 0,938 |
|  | **PEI-UPF*Smoking** | -0,196 | 0,187 | 0,294 |
|  | **PEI-UPF*Dietary Folate** | -0,105 | 0,840 | 0,901 |
|  | **PEI-UPF*Dietary Vit B_12_ intake** | 0,003 | 0,029 | 0,921 |
| 6 | **(Constant)** | 6,404 | 2,828 | 0,024 |
|  | **PEI-UPF** | 0,085 | 0,531 | 0,872 |
|  | **Age** | 0,016 | 0,081 | 0,848 |
|  | **Smoking status** | 1,108 | 0,987 | 0,262 |
|  | **Folic acid supplement intake** | -0,442 | 0,563 | 0,433 |
|  | **Multivitamin supplement intake** | 0,466 | 0,791 | 0,556 |
|  | **Dietary Folate intake** | -2,138 | 4,178 | 0,609 |
|  | **Dietary Vitamin B_12_ intake** | -0,055 | 0,151 | 0,716 |
|  | **PEI-UPF*Age** | 0,005 | 0,016 | 0,740 |
|  | **PEI-UPF*Smoking** | -0,209 | 0,187 | 0,264 |
|  | **PEI-UPF*Dietary Folate** | -0,073 | 0,839 | 0,931 |
|  | **PEI-UPF*Dietary Vit B_12_ intake** | 0,005 | 0,029 | 0,869 |
|  | **PEI-UPF*Multivitamin suppl use** | -0,232 | 0,153 | 0,130 |
| 7 | **(Constant)** | 8,981 | 3,405 | 0,009 |
|  | **PEI-UPF** | -0,431 | 0,653 | 0,510 |
|  | **Age** | 0,014 | 0,081 | 0,868 |
|  | **Smoking status** | 0,833 | 1,006 | 0,408 |
|  | **Folic acid supplement intake** | -2,951 | 1,934 | 0,128 |
|  | **Multivitamin supplement intake** | 0,528 | 0,792 | 0,505 |
|  | **Dietary Folate intake** | -2,443 | 4,179 | 0,559 |
|  | **Dietary Vitamin B_12_ intake** | -0,058 | 0,151 | 0,700 |
|  | **PEI-UPF*Age** | 0,006 | 0,016 | 0,728 |
|  | **PEI-UPF*Smoking** | -0,153 | 0,191 | 0,424 |
|  | **PEI-UPF*Dietary Folate** | -0,012 | 0,839 | 0,989 |
|  | **PEI-UPF*Dietary Vit B_12_ intake** | 0,006 | 0,029 | 0,835 |
|  | **PEI-UPF*Multivitamin suppl use** | -0,241 | 0,153 | 0,116 |
|  | **PEI-UPF*Folic acid suppl use** | 0,503 | 0,371 | 0,176 |

**Supplemental Table 4.** Baseline characteristics of the study population within the GB cut-off and outside the GB-cut-off.

|  | Within GB cut-off  *n* = 1532 | Outside GB cut-off  *n* = 294 | P-value |
| --- | --- | --- | --- |
| Age (years) | 32.5 [± 4.5] | 31.8 [± 5.0] | **.03** |
| BMI (kg/m^2^) | 24.1 [21.8-27.2] | 29.3 [24.9-33.1] | **<.001** |
| Geographical origin   - Western - Non-western - *Missing* | 1306 (88.0)  178 (12.0)  *48* | 219 (78.5)  60 (21.5)  *15* | **<.001** |
| Educational level   - Low - Middle - High - *Missing* | 112 (7.6)  496 (33.4)  875 (59.0)  *49* | 30 (10.8)  118 (42.3)  131 (47.0)  *15* | **<.001** |
| Smoking   - Yes - No - *Missing* | 217 (14.7)  1257 (85.3)  *58* | 39 (13.9)  241 (86.1)  *14* | 0.730 |
| Folic acid supplements   - Yes - No - *Missing* | 1458 (98.3)  25 (1.7)  *49* | 272 (97.1)  8 (2.9)  *14* | 0.224 |
| Multivitamin supplements   - Yes - No - *Missing* | 1059 (71.5)  423 (28.5)  *50* | 155 (55.8)  123 (44.2)  *16* | <.001 |
| tHcy (µmol/L) | 6.3 [5.3-7.3] | 6.5 [5.3-7.6] | 0.226 |
| Total intake (kJ/day) | 7881 [6733-9379] | 4746 [3890-5471] | **<.001** |
| PEI-UPF | 48.5 [40.6-56.4] | 46.0 [37.0-54.3] | **<.001** |

Values shown are mean (±SD) for age, median (IQR) for BMI, tHcy and total intake. Geographical origin, educational level, smoking and supplement use are presented as counts and percentages, n(%). P-values are calculated using ungrouped t-test or the Chi-square test.

**Supplemental Table 5.** Associations between periconceptional ultra-processed foods intake and tHcy in the study population including underreporters and stratification for BMI.

| All participants | β | 95% CI | p-value | n | Adjusted R Square |
| --- | --- | --- | --- | --- | --- |
| Basic model ^a^ | 0.18 | 0.10-0.27 | <.001 | 1826 | 0.009 |
| Basic model + MSAS ^b^ | 0.18 | 0.08-0.27 | <.001 | 1747 | 0.021 |
| Basic model + MSAS + all IT ^c^ | 0.82 | -0.01-1.64 | .05 | 1747 | 0.023 |
| Basic model + MSAS + selected IT ^d^ | 0.71 | -0.07-1.50 | .08 | 1747 | 0.023 |
|  | | | | | |
| BMI 18.5 – 24.9 kg/m^2 *^ | **β** | **95% CI** | **p-value** | **n** | **Adjusted R Square** |
| Basic model ^a^ | 0.22 | 0.09-0.35 | <.001 | 939 | 0.011 |
| Basic model + MSAS ^b^ | 0.23 | 0.08-0.37 | .002 | 905 | 0.016 |
| Basic model + MSAS + all IT ^c^ | 0.83 | -0.22-1.88 | .123 | 905 | 0.027 |
| Basic model + MSAS + selected IT ^d^ | 0.96 | 0.02-1.89 | <.05 | 905 | 0.031 |
|  | | | | | |
| BMI 25.0 – 29.9 kg/m^2 *^ | **β** | **95% CI** | **p-value** | **n** | **Adjusted R Square** |
| Basic model ^a^ | 0.16 | 0.04-0.29 | .01 | 521 | 0.011 |
| Basic model + MSAS ^b^ | 0.16 | 0.03-0.29 | .02 | 495 | 0.043 |
| Basic model + MSAS + all IT ^c^ | -0.05 | -0.99-0.88 | .91 | 495 | 0.050 |
| Basic model + MSAS + selected IT ^d^ | -0.16 | -1.07-0.76 | .73 | 495 | 0.040 |
|  | | | | | |
| BMI ≥ 30 kg/m^2 *^ | **β** | **95% CI** | **p-value** | **n** | **Adjusted R Square** |
| Basic model ^a^ | 0.05 | -0.15-0.25 | .62 | 334 | ֊0.002 |
| Basic model + MSAS ^b^ | 0.05 | -0.16-0.26 | .66 | 315 | 0.004 |
| Basic model + MSAS + all IT ^c^ | -0.11 | -1.55-1.32 | .87 | 315 | -0.005 |
| Basic model + MSAS + selected IT ^d^ | -0.24 | -1.58-1.10 | .73 | 315 | -0.002 |

^a^ Basic model: unadjusted. ^b^ Basic model + MSAS (minimal sufficient adjustment set of variables): Adjusted for MSAS identified in the DAG (directed acyclic graph): age, BMI, smoking status, folate and vitamin B_12_ dietary intake, folic acid and multivitamin supplement use. ^c^ Basic model + MSAS + all IT (interaction terms): adjusted for MSAS and all interaction terms. ^d^ Basic model + MSAS + selected IT: Adjusted for MSAS and significant interaction terms. ^*^In the analysis stratified for BMI: no adjustments were made for BMI and the interaction term PEI-UPF*BMI was excluded.
